# Endophytic plant growth promoting bacteria from two halophytes improve wheat performance under salt stress

**DOI:** 10.3389/fpls.2026.1658930

**Published:** 2026-01-27

**Authors:** Xuemin He, Hongfei Yuan, Yan Li, Chen Yang

**Affiliations:** 1 College of Ecology and Environment, Xinjiang University, Urumqi, China; 2 Key Laboratory of Oasis Ecology, Ministry of Education, Urumqi, China; 3 Technology Innovation Center for Ecological Monitoring and Restoration of Desert-Oasis, Ministry of Natural Resources, Urumqi, China; 4 Xinjiang Jinghe Observation and Research Station of Temperate Desert Ecosystem, Ministry of Education, Urumqi, China

**Keywords:** antioxidant enzymes, halophytes, PGPE, salt tolerance, wheat

## Abstract

Plant growth-promoting endophytes (PGPE) in halophytes have the potential to enhance plant stress resistance and promote growth, demonstrating broad application prospects in agriculture. The culturable microorganisms inhabiting in halophytes and their potential roles in enhancing salt-stress resistance of crops remain limited. This study isolated culturable endophytic bacteria from the roots of two dominant desert halophytes, *Haloxylon ammodendron* and *Halostachys caspica*, determined their growth-promoting abilities, and evaluated their capability in improving wheat performance under salt stress. Five saline-alkali tolerant bacterial strains—identified as *Priestia endophyticus* (S1, Y5), *Priestia licheniformis* (S2), *Streptomyces griseorubens* (S7), and *Nocardiopsis aegyptia* (Y6)—were characterized. These bacterial strains exhibited robust survival in 1.4 mol/L NaCl and high-pH environments (pH > 11.0), while demonstrating multiple growth-promoting traits, including indole-3-acetic acid (IAA) production and inorganic phosphate solubilization. All of the five strains (except for S2) and mixed culture improved the germination potential at 100 mmol/L NaCl. The strains S7, Y5, and mixed culture significantly increased plant height, root length, above ground fresh and dry weight compared to 200 mmol/L NaCl stressed seedlings (200CK)(*p* < 0.05). Salt stress significantly decreased chlorophyll content by 25.82% and 34.06% under 100 and 200 mmol/L NaCl in comparison to CK. Conversely, PGPE inoculation significantly promoted chlorophyll synthesis of seedlings under salt stress. PGPE inoculation reduced enzyme activities of peroxidase (POD), superoxide dismutase (SOD), and catalase (CAT) relative to the salt stressed seedlings. All inoculation treatments significantly decreased SOD activity by 20.2%–34.62%, and POD activity by 30.79%–53.38%, relative to 200CK. These findings demonstrate that these strains isolated from halophytic plants exhibit positive effects in ameliorating salt stress and improving the growth of wheat seedlings, highlighting their potential for enhancing agricultural productivity in saline-alkali soils.

## 1 Introduction

Soil salinization is a critical global issue, affecting over 800 million hectares or approximately 6% of the world’s total land (Munns and Tester, 2008). This problem is exacerbated by natural factors, such as climate change and specific geological conditions (Zhao et al., 2013). Additionally, unsustainable agricultural practices—including long-term monoculture, excessive fertilization, and poor-quality irrigation—further intensify salinization (Farahat et al., 2020). Excessive salt levels impair plant physiology via osmotic and ionic stress (Meng et al., 2018; Hao et al., 2021). These stressors reduce plant community diversity and lead to significant declines in crop productivity (Kramer and Mau, 2023; Tessema et al., 2023; Sharifuzzaman and Islam, 2024).

Halophytes, which comprise about 1%-2% of all terrestrial flora (Taulé et al., 2021), are capable of thriving in highly saline soils exceeding 250 mmol/L NaCl (Flowers and Colmer, 2008). They play a crucial role in stabilizing saline-alkali soils, particularly in desert ecosystems. These plants have evolved various tolerance mechanisms: some secrete salts out of the roots, others compartmentalize salts in central vacuole, and some secrete salts through specialized salt glands on their leaves (Hajibagheri and Flowers, 1989; Wang et al., 2006). The adaptation of halophytes to high-salinity environments is also closely linked to highly specific salt-tolerant endophytic and rhizosphere microorganisms (Santoyo et al., 2016; Etesami and Beattie, 2018). Endophytes—non-pathogenic bacteria and fungi that reside within plant tissues during part or all of their life cycle—are collectively referred to as plant endophytes (Santoyo et al., 2016).

Plant Growth-Promoting Endophytes (PGPE) are a category of microorganisms that inhabit internal plant tissues, such as roots, stems, and leaves, forming mutualistic relationships with their hosts (Compant et al., 2010). Most PGPE enhance host plant growth and nutrient acquisition, as well as improve plant tolerance to abiotic stresses (El-Esawi et al., 2017). These endophytes promote plant development through multiple mechanisms (Hardoim et al., 2015; Santoyo et al., 2016). They facilitate the absorption of essential nutrients, such as nitrogen and phosphorus (Roy et al., 2021). Furthermore, they activate antioxidant defenses, including superoxide dismutase (SOD), and induce the accumulation of osmoprotectants like proline and soluble sugars (Singh et al., 2020). Other beneficial functions include the production of extracellular polysaccharides and the secretion of ACC deaminase to mitigate stress. Additionally, they enhance plant stress tolerance through the synthesis of phytohormones like IAA, ABA, and volatile organic compounds (VOCs) which act as signaling molecules that modulate the expression of salt-responsive genes and transcription factors (Kaushal and Wani, 2016; Ilangumaran and Smith, 2017; Rosier et al., 2018). The application of microbial fertilizers containing plant growth-promoting microorganisms can reduce soil salinity and pH, optimize soil environmental quality, increase soil nutrient content, and enhance plant growth (Han et al., 2015) and crop yield (Injamum-Ul-Hoque et al., 2024; Li et al., 2024; Wei et al., 2024). Zafar-ul-Hye and colleagues reported that inoculation of PGPR is a promising strategy to mitigate salinity stress for improving wheat growth and yield (Zafar-Ul-Hye et al., 2019). Similarly, Prittesh et al. demonstrated that the salt tolerant PGPR strains from genera *Bacillus*, *Exiguobacterium*, *Enterobacter*, *Lysinibacillus*, *Stenotrophomonas*, and *Microbacterium* improved the growth and biomass production of rice under salinity stress (Prittesh et al., 2020).

In China, the soil salinization affects approximately 1.0×10^8^ hectares, with Xinjiang accounting for a significant portion, approximately 1.3×10^4^ hectares (Liang et al., 2022). This region is also rich in halophyte resources (Xiao and Zhou, 2023), including species such as *Suaeda salsa*, *S. glauca*, *Halostachys caspica*, and *Haloxylon ammodendron*, which adapt to saline-alkali environments through unique tolerance mechanisms and microbial synergies (Ondrasek et al., 2022). Recently, several strains affiliated to *Glutamicibacter halophytocola*, *Streptomyces* sp., *Streptomyces gardneri*, *Paenibacillus xylanexedenes*, *Enterobacter cloacae*, *Priestia*, *Arthrobacter agilis* have been isolated from halophytic plants, such as *Limonium sinensea*, *Salicornia* sp., *Pteropyrum olivieri*, *Phoenix dactylifera*, *Lycium ruthenicum*, *Halocnemum strobilaceum*, and *Tetragonia tetragonioides* (Yaish et al., 2015; Qin et al., 2017; Zhou et al., 2017; Liu et al., 2019; Xiong et al., 2020; Zahra et al., 2020; Gonçalves et al., 2021; Egamberdieva et al., 2022). Halophytes harbor a high abundance of PGPEs with great application prospects in plant growth promotion, biocontrol, stress resistance enhancement and crop yield improvement. Therefore, increased efforts are warranted to isolate and characterize salt-tolerant PGPEs from halophytes and elucidate their plant-beneficial functions.

*Halostachys caspica* (M. Bieb.) C. A. Mey. and *Haloxylon ammodendron* (C. A. Mey.) Bunge are two important halophytic plants in arid land. They have strong drought, and salt-alkali resistant ability, serving important ecological functions such as windbreaking and sand fixation, soil improvement, microclimate regulation, and biodiversity conservation. We previously investigated their root associated microbial composition by high-throughput sequencing approaches which provided insights into their potential mechanisms for salinity adaptation (Li et al., 2018). However, our understanding of culturable microorganisms and their potential roles in salinity resistance remains limited. Therefore, this study employed culture-dependent approach to: (1) isolate and identify endophytic bacteria from the two halophytic plants; (2) identify their plant growth-promoting abilities; and (3) assess their effect on enhancing wheat germination and seedling growth under salt stress.

## 2 Materials and methods

### 2.1 Root collection

Roots were collected from three healthy individuals of each plant species in the Ebinur Lake Area, a desert ecosystem of Northwestern China (**Supplementary Figure S1**). The roots were subjected to surface sterilization using the following protocol: they were first immersed in sterile water and sonicated for 15 minutes, followed by three rounds of sonication in sterile 1×PBS buffer (containing 136.89 mmol/L NaCl, 2.67 mmol/L KCl, 8.1 mmol/L Na_2_HPO_4_, 1.76 mmol/L KH_2_PO_4_, pH 7.4) for 15 minutes each to remove adhering soil particles. Subsequently, the roots were treated with 75% ethanol for *2–3 m*inutes, rinsed with sterile water, and sonicated again. Finally, they were soaked in 3.5% sodium hypochlorite (NaClO) solution for *4–5 m*inutes and thoroughly rinsed with sterile water (Li et al., 2020). The final rinse solution was collected, streaked onto solid culture medium, and incubated at 28°C for 2*4–48 h*ours to verify sterilization effectiveness. The absence of microbial growth confirmed successful surface sterilization, and the roots were then used for endophytic bacteria isolation.

### 2.2 Isolation, cultivation, and identification of endophytic bacteria

#### 2.2.1 Isolation and cultivation of roots endophytic bacteria

Bacteria were isolated according to the high-throughput isolation and cultivation protocol adapted from Zhang et al. (2021). Approximately 0.2 g of surface-sterilized roots were crushed separately using a sterile mortar with 25 mL of 10 mmol/L MgCl_2_ solution under aseptic conditions. The supernatant was diluted with 1 L of 10% TSB solution (containing 3 g/L TSB) and further diluted to three gradients (1/2 ODC, 1× ODC, and 2× ODC, where ODC denotes optimal dilution concentration) to enhance the success rate of single-bacterium isolation. Each dilution was transferred into ten 96-well cell culture plates (160 µL per well) and incubated at 30°C for two weeks. After incubation, 30 wells exhibiting diverse bacterial morphologies were selected from multiple plates for streak culture. Bacterial colonies were picked and transferred onto 1/2 TSB solid plates. After 3–5 days of incubation at room temperature, single colonies were subcultured onto fresh 1/2 TSB solid medium (containing 15 g/L TSB and 20 g/L agar). This purification process was repeated three times to obtain axenic cultures for subsequent identification.

#### 2.2.2 Identification of salt and alkali tolerance and plant growth-promoting abilities of strains

All isolated strains were inoculated onto 1/2 TSB solid medium supplemented with NaCl at final concentration of 0.34 mol/L (m/v 2%), 0.69 mol/L (4%), 1.03 mol/L (6%), 1.4 mol/L (8%), 1.7 mol/L (10%), and 2.1 mol/L (12%) (pH 7.5), respectively, and cultured at 30°C for 5 days. Colony formation was recorded as indicative of salt tolerance (+), while no growth was scored as intolerance (–). Salt-tolerant strains were further tested for alkali tolerance on media adjusted to pH 8.5, 9.0, 9.5, 10, 11, and 12, with the addition of 0.34 mol/L NaCl. Growth after 5 days at 30°C was recorded as alkali tolerance (+); absence of growth was marked as intolerance (–).

The phosphate-solubilizing ability was assessed using the molybdenum-antimony colorimetric method (Shukla et al., 2012). The indole-3-acetic acid (IAA) production ability was determined via the Salkowski colorimetric method (Glickmann and Dessaux, 1995). Siderophore production was evaluated on CAS medium (Schwyn and Neilands, 1987). Nitrogen-fixing ability was tested using the Ashby’s agar plate assay (Kim and Rees, 1994). ACC deaminase activity was determined according to the method proposed by Shurigin et al. (2020).

#### 2.2.3 Molecular identification of bacteria strains

The DNA was isolated using an Ezup Column Bacteria Genomic DNA Purification Kit (Sangon Biotech, Shanghai, China) according to the manufacturer’s protocols. The quality and concentration of DNA were tested using 1.5% agarose gel electrophoresis and Nanodrop Spectrophotometer (Thermo Fisher Scientific Inc., Waltham, MA, USA). The polymerase chain reaction (PCR) amplification of the 16S rRNA gene was performed using the 27F forward (AGAGTTTGATCMTGGCTCAG) and 1492R reverse (TACGGTACCTTGTTACGACTT) universal primers (Weisburg et al., 1991) by using an automated thermal cycler (Bio-rad, Hercules, California, USA) with the following PCR conditions: initial denaturation at 95°C for about 5 min followed by 30 cycles of amplification at 94°Cfor 30 s, annealing at 57°C for about 60 s, extension at 72°C for 90s, elongation at 72°C for 10 min. The PCR products were purified using a SanPrep column PCR product purification kit (Sangon Biotech., Shanghai, China) and quantified with a NanoDrop photometer. Purified amplicons were sequenced on an Applied Biosystems 3730XL Sequencer (Foster, CA, USA). The nucleotide sequences were edited and aligned using Chromas (v. 2.6.5) (http://technelysium.com.au/wp/chromas/), and compared with reference sequences in the NCBI GenBank database (http://www.ncbi.nlm.nih.gov/) using BLAST. The MEGA 11 software (Tamura et al., 2021) was used to construct the phylogenetic tree. The 16S rRNA sequences of the five endophytes were deposited in GenBank (NCBI) under the accession numbers PV687449 (S1), PV687450 (S2), PV687451 (S7), PV687452 (Y5), and PV687453 (Y6), respectively. The rRNA sequences were available in **Supplementary Material**.

### 2.3 The effects of endophytic bacteria on wheat germination and growth under salt stress

#### 2.3.1 Preparation of bacterial suspension

Five bacterial strains exhibiting high salt tolerance and plant growth-promoting ability (IAA production, phosphate solubilization, nitrogen fixation, siderophore production, etc.) were selected for inoculation experiments. Each strain was cultured in 1/2 TSB liquid medium at 30°C for 12 hours. The bacterial cells were harvested by centrifugation, washed, and resuspended in sterile water to a final concentration of 10^8^ CFU/mL (OD600 ≈ 1.0). A mixed bacterial suspension was prepared by combining equal volumes of the five individual suspensions. Thus, five single-strain suspensions and one mixed suspension were used in subsequent assays.

#### 2.3.2 Effects of growth-promoting bacteria on wheat seed germination under salt stress

To evaluate the impact of bacterial inoculation on seed germination under varying salinity, 15 treatments were implemented as described in **Table 1**.

**Table 1:** Experimental design for wheat seed germination assay under salt stress.

Treatment group		Inoculation treatment	NaCl concentration (mmol/L)
Control group	CK	Sterile water	0
Salt stress group	50CK/100CK	Sterile water	50/100
Salt stress + inoculation groups	50S1/100S1	*Priestia endophyticus* S1	50/100
	50S2/100S2	*Priestia licheniformis* S2	50/100
	50S7/100S7	*Streptomyces griseorubens* S7	50/100
	50Y5/100Y5	*Priestia endophyticus* Y5	50/100
	50Y6/100Y6	*Nocardiopsis aegyptia* Y6	50/100
	50Mix/100Mix	Mixed bacterial suspension	50/100

Wheat seeds were surface-sterilized by soaking in NaClO solution (effective chlorine content of 5%) for 3 minutes, rinsed thoroughly with sterile water, treated with 75% ethanol for 5 minutes, and rinsed again six times with sterile water (Younesikelaki et al., 2016). Seeds were immersed in bacterial suspensions for 24 hours; control seeds were soaked in sterile water. After treatment, seeds were placed on sterile Petri dishes lined with two layers of filter paper moistened with 10 mL of sterile water or NaCl solution (50 or 100 mmol/L). Each plate contained 12 seeds, with three plates per treatment. Germination was carried out at 25°C in darkness. Germination rate was evaluated on the seventh day, and germination potential was percentage of seeds that sprouted within the first 3 days.

#### 2.3.3 Effects of growth-promoting bacteria on wheat seedlings growth under salt stress

Fifteen treatments were designed to assess the efficacy of endophytes under 100 mmol/L and 200 mmol/L NaCl stress, as summarized in **Table 2**.

**Table 2:** Experimental design for wheat seedling growth assay under salt stress.

Treatment group		Inoculation treatment	NaCl concentration (mmol/L)
Control group	CK	Sterile water	0
Salt stress group	100CK/200CK	Sterile water	100/200
Salt stress + inoculation groups	100S1/200S1	*Priestia endophyticus* S1	100/200
	100S2/200S2	*Priestia licheniformis* S2	100/200
	100S7/200S7	*Streptomyces griseorubens* S7	100/200
	100Y5/200Y5	*Priestia endophyticus* Y5	100/200
	100Y6/200Y6	*Nocardiopsis aegyptia* Y6	100/200
	100Mix/200Mix	Mixed bacterial suspension	100/200

The seedling growth assay was conducted in a controlled environment to ensure consistent soil moisture and prevent waterlogging. Seeds were sterilized and pre-germinated as described above. Uniformly germinated seeds were transplanted into pots (220 × 160 mm) containing 1 kg of sterile soil (sand: garden soil = 1:1) that were sterilized through two rounds of autoclaving (30 min at 121°C). Three seedlings were sown to each pot, and placed in an artificial climate chamber (GZD-450B, LISK Instrument, Nanjing, China). The growth conditions were set to a constant temperature of 25°C, a light intensity of 10000 lux, and a light/dark ratio of 12 h/12 h for the first 7 days, followed by 14 h/10 h. Soil water content was set to about 70% of field water capacity, then maintained using the gravimetric method. After one week of seedlings growth, we conducted inoculation and salt treatments. 30 mL of bacterial suspension was applied twice per week for the inoculation groups, while the control groups (CK) and uninoculated + salt stress groups (100CK and 200CK) received an equal volume of sterile water. For salt stress, 50 mL of NaCl solution (100 or 200 mmol/L) were added into pot every two days for salt stress groups (100CK and 200CK) and salt stress + inoculation groups, while the CK received 50 mL of sterile water (Han et al., 2015).

After 30 days, seedlings were harvested for analysis of physiological parameters and antioxidant enzyme activities. Fresh weight was recorded immediately; samples were then dried at 105°C for 30 minutes and at 70°C for 48 hours to determine dry weight. Chlorophyll was extracted from fresh leaf samples (0.1 g) using 96% ethanol, and absorbance was measured at 649, 479, and 665 nm with a 723N spectrophotometer (Jinghua Technology). Chlorophyll content was calculated as described by Gao (2018). For enzyme assays, fresh leaves (0.1 g) were homogenized on ice, and the supernatant was used to determine peroxidase (POD), superoxide dismutase (SOD), and catalase (CAT) activities using commercial assay kits (Sangon Biotech, Shanghai, China) according to the manufacturer**’**s protocols. All measurements were performed in triplicate.

### 2.4 Statistical analysis

Data analysis was performed in R (v4.3.1). The normality (Shapiro-Wilk test) and homogeneity of variances (Levene*’*s test) were assessed first. For data meeting both assumptions, one-way ANOVA was conducted using aov(), followed by Tukey*’*s HSD *post-hoc* test for pairwise comparisons. For data violating either assumption, the Kruskal-Wallis test was used, followed by Dunn*’*s test with Bonferroni adjustment. A *p*-value < 0.05 was considered significant.

## 3 Results

### 3.1 Identification of salt-alkaline tolerance and growth-promoting abilities of isolated bacteria

After screening, five strains exhibiting high salt and alkaline tolerance—designated S1, S2, S7, Y5, and Y6—were obtained (**Supplementary Figures S2**, **S3**). Strains S1, S2, and S7 were isolated from the roots of *Haloxylon ammodendron*, while Y5 and Y6 originated from *Halostachys caspica* roots. All five strains were capable of surviving in a medium containing 2.1 mol/L NaCl (**Table 3**) and grew effectively in media with pH values exceeding 11.0 in the presence of 0.34 mol/L NaCl (**Table 4**).

**Table 3:** Salt tolerance determination of bacterial strains.

Strain number	Host	Salinity (mol/L NaCl, Medium pH 7.5)					
		0.34	0.69	1.03	1.4	1.7	2.1
S1	*H. ammodendron*	++	++	++	++	++	+
S2	*H. ammodendron*	++	++	++	++	++	+
S7	*H. ammodendron*	++	++	++	++	+	+
Y5	*H. caspica*	++	++	++	++	++	+
Y6	*H. caspica*	++	++	++	++	+	+

“++” represents good growth; “+” represents growth;”-” indicates no bacterial growth.

**Table 4:** Alkaline tolerance determination of bacterial strains.

Strain number	Host	pH (Medium salnity 0.34 mol/L NaCl)					
		8.5	9	9.5	10	11	12
S1	*H. ammodendron*	++	++	++	++	+	+
S2	*H. ammodendron*	++	++	++	++	++	+
S7	*H. ammodendron*	++	++	++	+	+	–
Y5	*H. caspica*	++	++	++	++	+	+
Y6	*H. caspica*	++	++	++	++	+	+

“++” represents good growth; “+” represents growth;”-” indicates no bacterial growth.

All strains had nitrogenase activity (**Supplementary Figure S4**) and produced indole-3-acetic acid (IAA), with S2 yielding the highest IAA concentration of 24.8 mg/L (*p* < 0.05). With the exception of Y6, all strains exhibited inorganic phosphate solubilization capacity. Notably, Y5 showed the highest phosphate solubilization ability, reaching 146.17 mg/L, which was significantly greater than that of the other strains (*p* < 0.05). Siderophore production was observed in strains S7 and Y6, while ACC deaminase activity was detected in S1, S2, Y5, and Y6 (**Table 5**).

**Table 5:** The plant growth promoting capability of the five endophytic bacterial strains.

Strain number	Nitrogen fixation	P- solubilization (mg/L)	IAA(mg/L)	Siderophore	ACC deaminase
S1	+	98.36 ± 5.50b	8.43 ± 1.27b	–	+
S2	+	108.92 ± 5.30b	24.80 ± 1.67a	–	+
S7	+	64.5 ± 7.52c	2.64 ± 0.47d	+	–
Y5	+	146.17 ± 5.82a	3.63 ± 0.10cd	–	+
Y6	+	–	5.92 ± 0.65bc	+	+

*“*+*”*: The function is available. *“*-*”*: This function is not available. Different letters indicate significant differences at 0.05 level, data represented by mean ± standard error (n = 3).

### 3.2 Identification of bacterial species

**Figure 1:**
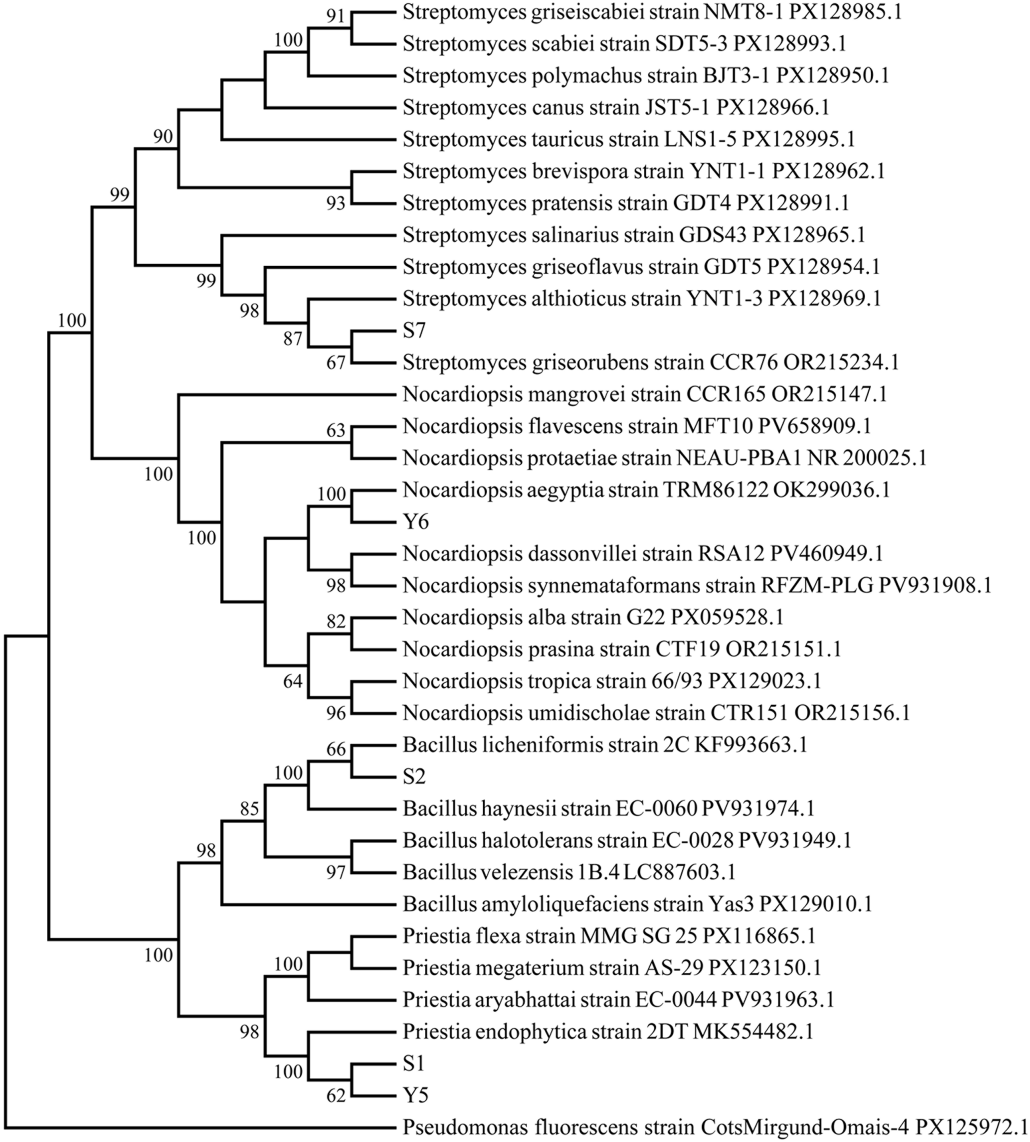
Neighbor-Joining phylogenetic tree constructed with 16S rDNA gene sequences. Taxon labels include scientific name, strain number and Genbank accesion number, except for strains S1, S2, S7, Y5, and Y6.

The 16S rDNA sequences of the five root endophytes (S1, S2, S7, Y5, and Y6) ranged from 1,313 to 1,488 bp in length. Sequence comparisons with the GenBank database revealed identity percentages between 99.18% and 100% with the closest related strains. A phylogenetic tree constructed using the Neighbor-Joining method illustrated the relationships between the isolated endophytes and their closest relatives (**Figure 1**). The strains were identified as *Priestia endophyticus* S1, *Priestia licheniformis* S2, *Priestia endophyticus* Y5, *Streptomyces griseorubens* S7, and *Nocardiopsis aegyptia* Y6.

### 3.3 Effects of growth-promoting bacteria on wheat performance under salt stress

#### 3.3.1 Effects on seed germination

**Figure 2:**
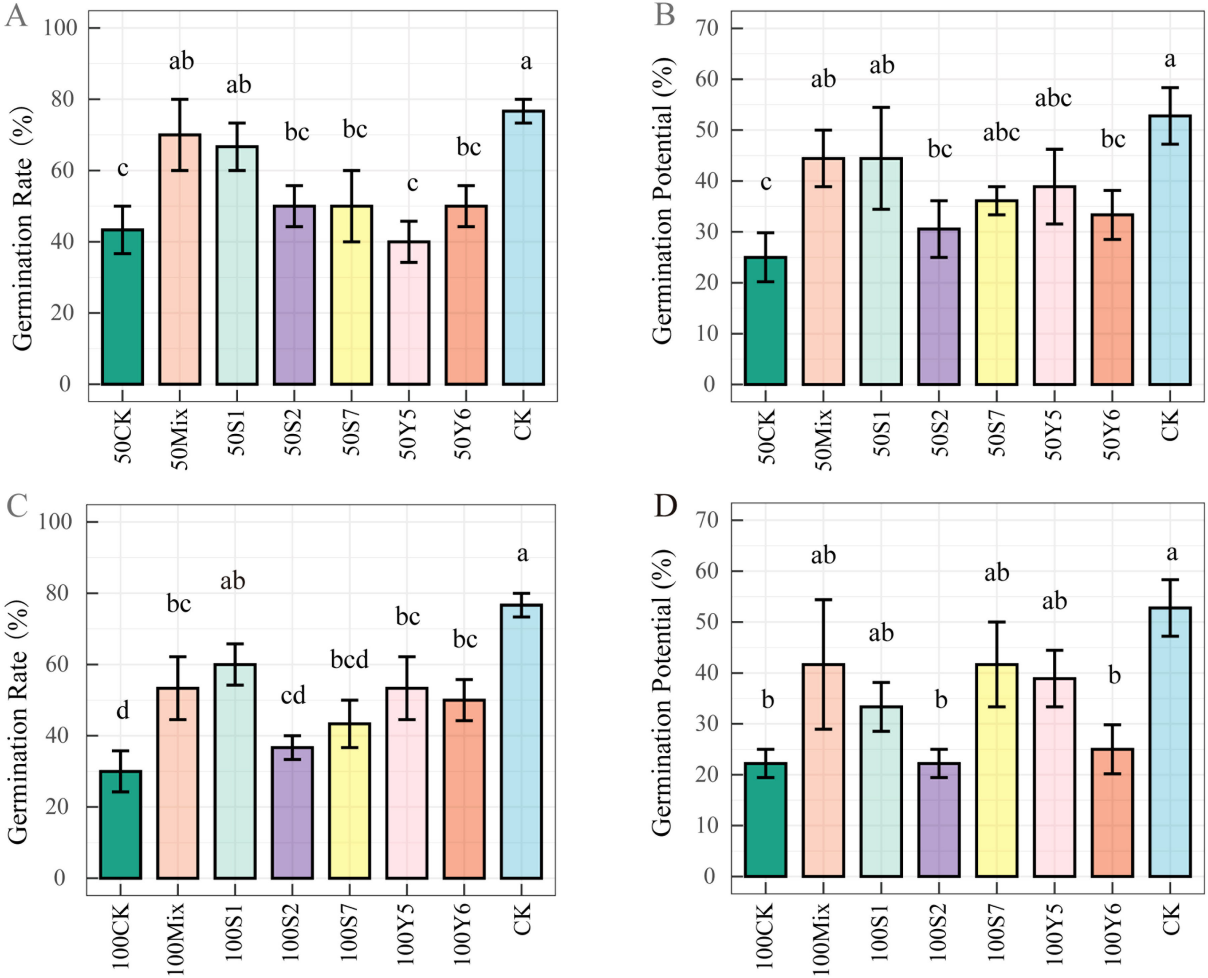
Effects of endophytic bacteria inoculation on wheat seeds germination under salt stress. Error bars represent standard error (n = 10). Different letters indicate significant differences at *p* < 0.05. **(A, B)** germination rate; **(C, D)** germination potential. Abbreviations: CK, untreated seeds irrigated with sterile water; 50CK, seeds treated with 50 mmol/L NaCl; 100CK, seeds treated with 100 mmol/L NaCl; 50S1, seeds inoculated with strain S1 and 50 mmol/L NaCl; 100S1, seeds inoculated with strain S1 and 100 mmol/L NaCl; 50S2, seeds inoculated with strain S2 and 50 mmol/L NaCl; 100S2, seeds inoculated with strain S2 and 100 mmol/L NaCl; 50S7, seeds inoculated with strain S7 and 50 mmol/L NaCl; 100S7, seeds inoculated with strain S7 and 100 mmol/L NaCl; 50Y5, seeds inoculated with strain Y5 and 50 mmol/L NaCl; 100Y5, seeds inoculated with strain Y5 and 100 mmol/L NaCl; 50Y6, seeds inoculated with strain Y6 and 50 mmol/L NaCl; 100Y6, seeds inoculated with strain Y6 and 100 mmol/L NaCl; 50Mix, seeds inoculated with mixed bacterial suspension and 50 mmol/L NaCl; 100Mix, seeds inoculated with mixed bacterial suspension and 100 mmol/L NaCl. Corresponding visual representations are provided in **Supplementary Figures S5** and **S6**.

Five endophytic strains (S1, S2, S7, Y5, and Y6) and their mixed inoculant were evaluated for their ability to alleviate salt stress during wheat seed germination and seedling growth. Salt stress significantly reduced the germination of wheat seeds (*p* < 0.05, **Figure 2**). Under 50 mmol/L NaCl, strain S1 and the mixed culture significantly increased the germination rate and germination potential compared to 50CK (*p* < 0.05, **Figures 2A, B**). At 100 mmol/L NaCl, the germination rate of S1 and mixed culture inoculation were 60.0% and 58.0%, respectively, significantly higher than that of 100CK (30%, *p* < 0.05). Furthermore, all of the five strains (except for S2) and mixed culture improved the germination potential compared to 100CK, though not significant (**Figures 2C, D**). These indicated that bacterial inoculation improved wheat seed germination under salt stress.

#### 3.3.2 Effects on seedling biomass and chlorophyll content

Salt stress (100 and 200 mmol/L NaCl) severely inhibited seedling development, significantly reducing plant height, root length, and chlorophyll content relative to CK (**Supplementary Figures S5**, **S6**). Bacterial inoculation effectively mitigated these inhibitory effects (**Supplementary Figure S7**). Under 100 mmol/L NaCl, strains S7, Y5, and Y6 significantly increased plant height and root length relative to 100CK (*p* < 0.05, **Table 6**). The inoculation also alleviated salt stress and improved seedling growth under 200 mmol/L NaCl. The strains S7, Y5, and mixed culture significantly increased plant height, root length, above ground fresh and dry weight compared to 200CK (*p* < 0.05, **Table 7**). Strain S7 increased the plant height, root length, above ground fresh and dry weight by 3.8%, 3.39%, 43.2%, and 51.9% respectively more than the uninoculated seedlings; The plant height, root length, above ground fresh and dry weight were 6.4%, 27.2%, 45%, and 46.2% higher in strain Y5 inoculated seedlings than those of 200CK respectively; Mixed strain inoculation increased these parameters by 9.9%, 31.8%, 55%, and 48.1% respectively compared to 200CK.

**Table 6:** Effects of PGPE inoculation on wheat seedlings physiology under 100 mmol/L NaCl stress.

Treatment	Plant height (cm)	Root length (cm)	Above-ground fresh weight(g)	Above-ground dry weight (g)	Chlorophyll content (mg·g^-1^ FW)
CK	43.66 ± 1.09a	17.75 ± 2.26a	3.18 ± 0.22b	0.90 ± 0.07ab	5.44 ± 0.06a
100CK	38.70 ± 0.66c	11.88 ± 1.73c	3.06 ± 0.24b	0.75 ± 0.06b	4.03 ± 0.70c
100Mix	41.61 ± 1.04b	14.13 ± 0.76bc	3.04 ± 0.44b	0.81 ± 0.06ab	4.12 ± 0.33c
100S1	42.67 ± 1.22ab	13.75 ± 1.11bc	3.59 ± 0.50ab	0.95 ± 0.13ab	5.12 ± 0.12ab
100S2	42.11 ± 1.14ab	13.92 ± 1.17bc	2.98 ± 0.23b	0.86 ± 0.06ab	4.90 ± 0.19abc
100S7	41.44 ± 1.09b	15.78 ± 0.93ab	3.15 ± 0.04b	0.82 ± 0.04ab	4.71 ± 0.23abc
100Y5	40.97 ± 1.20b	14.65 ± 0.49b	4.24 ± 0.22a	1.03 ± 0.08a	4.87 ± 0.14abc
100Y6	41.10 ± 1.15b	14.55 ± 1.19b	3.19 ± 0.27b	0.86 ± 0.07ab	4.39 ± 0.24bc

Data shown in mean ± standard error (n=3). Different letters indicate significant differences at *p* < 0.05 among treatments. Abbreviations: CK, untreated seedlings irrigated with sterile water; 100CK, seedlings treated with 100 mmol/L NaCl; 100S1, seedlings inoculated with strain S1 under 100 mmol/L NaCl; 100S2, seedlings inoculated with strain S2 under 100 mmol/L NaCl; 100S7, seedlings inoculated with strain S7 under 100 mmol/L NaCl; 100Y5, seedlings inoculated with strain Y5 under 100 mmol/L NaCl; 100Y6, seedlings inoculated with strain Y6 under 100 mmol/L NaCl; 100Mix, seedlings inoculated with mixed strains under 100 mmol/L NaCl.

**Table 7:** The effects of PGPE inoculation on wheat seedlings physiology under 200 mmol/L NaCl stress.

Treatment	Plant height (cm)	Root length (cm)	Above-ground fresh weight(g)	Above-ground dry weight (g)	Chlorophyll content (mg·g^-1^ FW)
CK	43.66 ± 1.09a	17.75 ± 2.26a	3.18 ± 0.22a	0.90 ± 0.07a	5.44 ± 0.06a
200CK	37.06 ± 1.13d	10.87 ± 1.10c	2.20 ± 0.28b	0.52 ± 0.04c	3.59 ± 0.15d
200Mix	40.72 ± 0.91b	14.33 ± 0.96b	3.41 ± 0.36a	0.77 ± 0.05ab	4.08 ± 0.37cd
200S1	40.67 ± 1.02b	13.1 ± 0.81bc	2.80 ± 0.31ab	0.68 ± 0.10bc	4.79 ± 0.03b
200S2	38.24 ± 0.77cd	13.25 ± 1.12bc	2.31 ± 0.10b	0.66 ± 0.01bc	4.564 ± 0.09bc
200S7	38.47 ± 0.62cd	14.55 ± 1.67b	3.15 ± 0.21a	0.79 ± 0.05ab	4.656 ± 0.19b
200Y5	39.44 ± 1.08bc	13.83 ± 0.65b	3.19 ± 0.33a	0.76 ± 0.09ab	4.772 ± 0.11b
200Y6	38.09 ± 0.60cd	14.50 ± 1.24b	2.34 ± 0.14b	0.69 ± 0.08bc	4.696 ± 0.11b

Data shown in mean ± standard error (n=3). Different letters indicate significant differences at *p* < 0.05 among treatments. Abbreviations: CK, untreated seedlings irrigated with sterile water; 200CK, seedlings treated with 200 mmol/L NaCl; 200S1, seedlings inoculated with strain S1 under 200 mmol/L NaCl; 200S2, seedlings inoculated with strain S2 under 200 mmol/L NaCl; 200S7, seedlings inoculated with strain S7 under 200 mmol/L NaCl; 200Y5, seedlings inoculated with strain Y5 under 200 mmol/L NaCl; 200Y6, seedlings inoculated with strain Y6 under 200 mmol/L NaCl; 200Mix, seedlings inoculated with mixed strains under 200 mmol/L NaCl.

Chlorophyll content decreased by 25.82% and 34.06% under 100 and 200 mmol/L NaCl stress in comparison to CK, respectively. Conversely, PGPE inoculation significantly promoted chlorophyll synthesis. Under 100 mmol/L NaCl treatment, the chlorophyll content in wheat increased by 26.85%, 21.42%, 16.81%, 20.68%, and 8.75% upon inoculation with S1, S2, S7, Y5, and Y6, respectively (**Table 6**). Under 200 mmol/L NaCl, all five strains significantly enhanced chlorophyll content by 33.4%, 27.1%, 29.7%, 32.9%, 30.8%, respectively compared to 200CK (*p* < 0.05; **Table 7**). These demonstrated a positive effect on chlorophyll accumulation and photosynthesis.

#### 3.3.3 Effects on antioxidant enzyme activities

**Figure 3:**
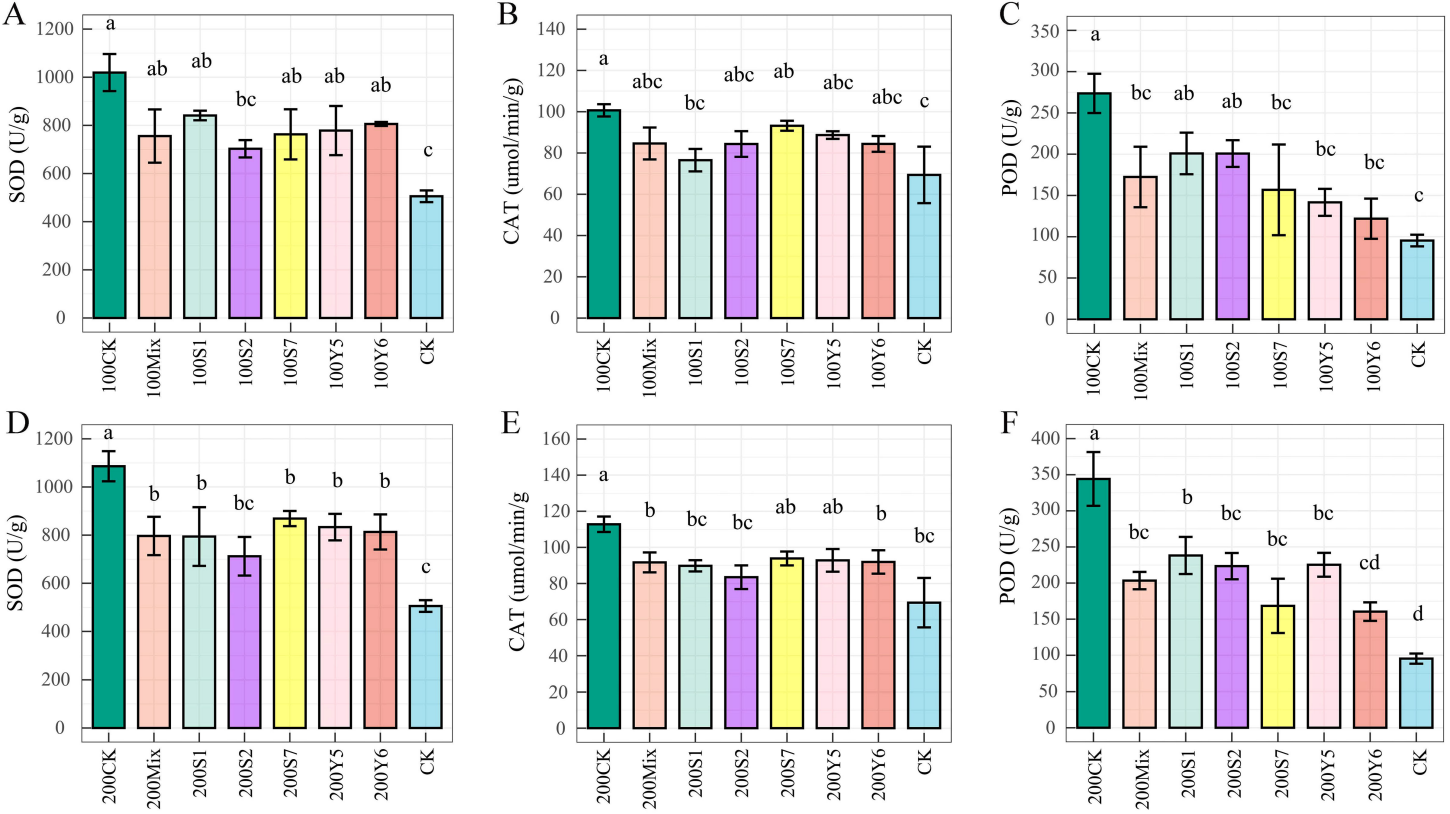
Effect of plant growth-promoting bacteria on antioxidant enzyme activity in wheat seedlings under salt stress. **(A–C)** represent treatments under 100 mmol/L NaCl. **(D–F)** represent treatments under 200 mmol/L NaCl. Abbreviations: CK, untreated seedlings irrigated with sterile water; 100CK, seedlings treated with 100 mmol/L NaCl; 200CK, seedlings treated with 200 mmol/L NaCl; 100S1, seedlings inoculated with strain S1 under 100 mmol/L NaCl; 100S2, seedlings inoculated with strain S2 under 100 mmol/L NaCl; 100S7, seedlings inoculated with strain S7 under 100 mmol/L NaCl; 100Y5, seedlings inoculated with strain Y5 under 100 mmol/L NaCl; 100Y6, seedlings inoculated with strain Y6 under 100 mmol/L NaCl; 100Mix, seedlings inoculated with mixed strains under 100 mmol/L NaCl; 200S1, seedlings inoculated with strain S1 under 200 mmol/L NaCl; 200S2, seedlings inoculated with strain S2 under 200 mmol/L NaCl; 200S7, seedlings inoculated with strain S7 under 200 mmol/L NaCl; 200Y5, seedlings inoculated with strain Y5 under 200 mmol/L NaCl; 200Y6, seedlings inoculated with strain Y6 under 200 mmol/L NaCl; 200Mix, seedlings inoculated with mixed strains under 200 mmol/L NaCl.

Salt stress induced a sharp increase in antioxidant enzyme activities under 100 and 200 mmol/L NaCl stress compared to CK. For instance, superoxide dismutase (SOD) activity increased by 101% and 146% relative to CK, peroxidase (POD) activity increased by 186% and 260%, while catalase (CAT) activity increased by 45.1% and 62.25% (**Figure 3**). However, PGPE inoculation reduced these activities relative to the 100CK and 200CK, indicating reduced oxidative damage. Under 200 mmol/L NaCl stress, all inoculation treatments significantly decreased SOD activity by 20.2%–34.62% relative to 200CK, with strain S2 and the mixed inoculant showing the most pronounced effects (**Figures 3A, D**). Strain S1 inoculation decreased CAT activity by 24.03% and 20.39% relative to 100CK and 200CK, respectively (*p* < 0.05); Strains S7 and Y5 inoculation also reduced CAT activity, even the effects were not statistically significant (**Figures 3B, E**). Inoculation resulted in dramatic reductions; at 200 mmol/L NaCl, all strains significantly decreased POD activity by 30.79%–53.38% relative to 200CK, with strain Y6 exhibiting the highest efficacy (**Figures 3C, F**).

## 4 Discussion

Through long-term adaptation to saline-alkaline environments, halophytes have evolved specific salt tolerance mechanisms and developed unique associations with endophytic microorganisms. Previous studies have revealed that the most dominant genera of salt-tolerant bacteria in various halophytes include *Halomonas*, *Bacillus*, *Streptomyces*, *Oceanobacillus* and *Pseudomonas* (Etesami and Glick, 2020). These endophytic microorganisms improve the adaptability of plants to alkaline environments and maintain normal growth and development through diverse mechanisms (Egamberdieva et al., 2022). In this study, we isolated five endophytic bacterial strains belonging to the genera *Priestia*, *Streptomyces* and *Nocardiopsis*. Consistent with previous studies, these endophytic bacteria strains exhibited multiple plant growth-promoting traits, including the production of phytohormones, iron carriers, phosphate solubilization, nitrogenase activity, and ACC deaminase (Cho et al., 2015; Liu et al., 2019; Soldan et al., 2019; Babar et al., 2021).

Among the five strains, *Priestia endophyticus* Y5 shows the highest phosphorus-solubilizing ability (up to 146.17 mg/L), followed by *P. endophyticus* S1*, Streptomyces griseorubens* S7, and *P. licheniformis S2. P. endophyticus* is an aerobic, Gram-positive, nonmotile, rod-shaped, endospore-forming bacterium first isolated from the inner tissues of cotton plants (Reva et al., 2002). Certain strains of this species are known to confer pathogen-resistance by antibiotic production (Socha et al., 2007) and alleviate drought stress via exopolysaccharide secretion. The strong phosphorus-solubilizing ability of strain Y5 indicates that different *P. endophyticus* strains may vary in their plant growth-promoting capacities.

The strain S2, isolated from *H. ammodendron*, showed the highest indole-3-acetic acid (IAA) production among the five isolates. This indicates that *P. licheniformis* can colonize a range of plant species and function as an effective plant growth promoter. Previous studies have isolated *P. licheniformis* from the rhizosphere or roots of *Vigna radiata*, *Chenopodium quinoa*, and *Suaeda fruticosa*, noting its abilities to produce IAA, siderophores, ammonia, organic acids, and hydrogen cyanide, as well as to solubilize phosphate (Goswami et al., 2014; Mahdi et al., 2020; Bhutani et al., 2022).

Iron-carrier-producing strains play a positive role in improving the absorption and utilization efficiency of iron elements by plants and inhibiting the growth and reproduction of harmful microorganisms (Xiong et al., 2020). We find that *S. griseorubens* S7 and *Nocardiopsis aegyptia* Y6 exhibite siderophore production ability. Previous studies have reported that *S. griseorubens* have antimicrobial activities (Baygar and Ugur, 2017) and participate in polysaccharides degradation, such as xylan, chitin, and cellulose (Prasad et al., 2013; Feng et al., 2015; Meriem and Mahmoud, 2017). We additionally find that this strain also produces IAA and shows high P-solubilization activity, although it was the only one among the five strains that lacked ACC deaminase activity. All five strains demonstrated nitrogen fixation ability, which facilitates plant nitrogen absorption and supports growth and yield. These findings broaden the understanding of the plant growth-promoting (PGP) traits of these endophytic bacteria and highlight their potential applications in agriculture and ecological restoration.

Plant growth-promoting bacteria can enhance plants’ resistance to environmental stress and improve their growth (Liu et al., 2021). In this study, through inoculation experiments, we further validated the salt-tolerant plant growth-promoting effects of the screened bacteria on wheat. Under salt stress, the root length, shoot length, aboveground fresh and dry weight of wheat were reduced across all salt concentrations. In contrast, inoculation with PGPE improved seed germination rate, potential, and overall plant growth. In particular, *P. endophyticus* Y5 increased plant height, root length, above ground fresh and dry weight of wheat seedlings up to 6.4%, 27.2%, 45%, and 46.2%, respectively, under 200 mmol/L NaCl stress. Additionally, the isolated strains increased the chlorophyll content of plants under salt stress. A higher chlorophyll content is advantageous for plants to maintain normal photosynthesis under stress, thereby enhancing their ability to resist salt stress. These results corroborate that endophytic bacteria can effectively promote the growth of various crops under salt stress and other adverse environmental conditions (Ding et al., 2017; Choudhury et al., 2021; Dif et al., 2022; Khan et al., 2022a; Mohamad et al., 2022; Asif et al., 2023).

The antioxidant enzymes CAT, POD, and SOD play a crucial role in protecting plant cells from oxidative damage. Both 100 mmol/L and 200 mmol/L NaCl stress increased CAT, POD, and SOD activities compared to the non-stressed control. Inoculation with single or mixed bacterial strains reduced the activities of SOD, POD, and CAT compared to uninoculated salt-stressed seedlings. For instance, wheat inoculated with strain S1 showed decreased CAT activity under various salt treatments, while *N. aegyptia* Y6 significantly reduced POD activity under salt stress. Similar findings have been reported by previous studies (Zhao et al., 2017; Zhu et al., 2024). Furthermore, strain *P. licheniformis* S2 reduced SOD activity. Consistent with our results, Kadmiri et al. observed that inoculation with *Pseudomonas fluorescens* Ms-01 lowered SOD activity in salt-stressed wheat, thereby improving defense responses (Kadmiri et al., 2018). These results indicate that the application of these bacteria alleviates salt stress for the wheat seedlings. The observed reduction in SOD, POD, and CAT activities in inoculated plants—compared to the highly elevated levels in salt-stressed controls—suggests that the endophytic bacteria effectively mitigated the initial oxidative strain. By reducing the accumulation of reactive oxygen species (ROS) through other mechanisms (such as improved osmotic adjustment or ion homeostasis), the bacteria lowered the plant’s requirement for massive antioxidant enzyme production, thereby preventing potential secondary damage from prolonged high enzyme activity and conserving metabolic energy for growth.

While some studies have reported that bacterial consortia are more effective than single strains in promoting plant growth under stress, for example, Khan et al. (2022b) found that the application of strain composite inoculants significantly increased wheat growth compared to single-strain inoculation, further optimizing and improving the effect. Ahmad et al. (2012) reported that combined application of *Rhizobium phaseoli* and *Pseudomonas* strains was more effective in improving the productivity of mung beans under salt stress conditions compared to single-strain inoculation. However, we found that the mixed inoculation did not outperform the best single strains (S1 and Y5) in terms of enhancing shoot fresh weight or leaf chlorophyll content. These differences underscore the need for further optimization of bacterial combinations and ratios to maximize growth-promoting efficacy.

In summary, the root endophytes of *H. ammodendron* and *H. caspica* represent a valuable resource for salt-tolerant plant growth-promoting bacteria. The five strains isolated in this study demonstrated significant potential in improving salt tolerance and promoting growth in wheat through various mechanisms. Future work should focus on isolating more microbial strains, optimizing culture conditions, dilution concentrations, and consortium formulations to better mitigate salt stress and improve crop growth and yield.

## 5 Conclusions

This study identifies five saline-alkali tolerant endophytes with multiple PGP traits from desert halophytes. These bacterial strains exhibited robust survival ability in saline and alkali environment, as well as multiple growth-promoting traits, including indole-3-acetic acid (IAA) production and inorganic phosphate solubilization. Inoculation with these strains, particularly *Priestia endophyticus* S1 for enhancing germination and *P. endophyticus* Y5 for improving seedling biomass and chlorophyll synthesis, significantly alleviated the deleterious effects of salinity on wheat. The bacteria-mediated reduction in antioxidant enzyme activities (SOD, POD, and CAT) relative to salt stressed seedlings which indicates reduced level of oxidative stress in inoculated plants. These findings demonstrate the high potential of halophyte-derived endophytes as effective bio-fertilizers for improving crop productivity and land-use efficiency in saline-alkali regions.

## Data Availability

The datasets presented in this study can be found in online repositories. The names of the repository/repositories and accession number(s) can be found below: https://www.ncbi.nlm.nih.gov/nuccore/, PV687449–PV687453.
