# Supplementary material for: Endophytic plant growth promoting bacteria from two halophytes improve wheat performance under salt stress
Source: Front Plant Sci. 2026 Jan 27;17:1658930. doi: 10.3389/fpls.2026.1658930 (PMC12887591; doi:10.3389/fpls.2026.1658930)
Supplement: Supplementary file 1 [file DataSheet1.docx]

Supplementary Material

# Supplementary Data

The 16S rDNA sequences of the five endophytic bacteria strains.

>*Priestia endophyticus* S1

GGCTCAGGACGAACGCTGGCGGCGTGCCTAATACATGCAAGTCGAGCGGAGTTTTGAAAAGCTTGCTTTTCAAAACTTAGCGGCGGACGGGTGAGTAACACGTGGGCAACCTGCCCTTGAGACGGGGATAACTCCGGGAAACCGGAGCTAATACCGGATAACACATATCTTCGCATGAGGATATGTTAGAAGGTGGCTTTTAGCTACCACTCAAGGATGGGCCCGCGGCGCATTAGCTAGTTGGTGAGGTAACGGCTCACCAAGGCGACGATGCGTAGCCGACCTGAGAGGGTGATCGGCCACACTGGGACTGAGACACGGCCCAGACTCCTACGGGAGGCAGCAGTAGGGAATCTTCCGCAATGGACGAAAGTCTGACGGAGCAACGCCGCGTGAGTGATGAAGGTTTTCGGATCGTAAAGCTCTGTTGTTAGGGAAGAACAAGTACCTGTTGAATAAGCAGGTACCTTGACGGTACCTAACCAGAAAGCCACGGCTAACTACGTGCCAGCAGCCGCGGTAATACGTAGGTGGCAAGCGTTGTCCGGAATTATTGGGCGTAAAGCGCGCGCAGGCGGTTCCTTAAGTCTGATGTGAAAGCCCACGGCTCAACCGTGGAGGGTCATTGGAAACTGGGGAACTTGAGTGCAGAAGAGGAGAGCGGAATTCCACGTGTAGCGGTGAAATGCGTAGAGATGTGGAGGAACACCAGTGGCGAAGGCGGCTCTCTGGTCTGTAACTGACGCTGAGGCGCGAAAGCGTGGGGAGCGAACAGGATTAGATACCCTGGTAGTCCACGCCGTAAACGATGAGTGCTAAGTGTTAGAGGGTTTCCGCCCTTTAGTGCTGCAGCAAACGCATTAAGCACTCCGCCTGGGGAGTACGGTCGCAAGACTGAAACTCAAAGGAATTGACGGGGACCCGCACAAGCGGTGGAGCATGTGGTTTAATTCGAAGCAACGCGAAGAACCTTACCAGGTCTTGACATCCTCTGCTACTTCTAGAGATAGAAGGTTCCCTTCGGGGACAGAGTGACAGGTGGTGCATGGTTGTCGTCAGCTCGTGTCGTGAGATGTTGGGTTAAGTCCCGCAACGAGCGCAACCCTTGATCTTAGTTGCCAGCATTTAGTTGGGCACTCTAAGGTGACTGCCGGTGACAAACCGGAGGAAGGTGGGGATGACGTCAAATCATCATGCCCCTTATGACCTGGGCTACACACGTGCTACAATGGATGATACAAAGGGCTGCGAAACCGCGAGGTTAAGCCAATCCCATAAAATCATTCTCAGTTCGGATTGTAGGCTGCAACTCGCCTACATGAAGCCGGAATCGCTAGTAATCGCGGATCAGCATGCCGCGGTGAATACGTTCCCGGGTCTTGTACACACCGCCCGTCACACCACGAGAGTTTGTAACACCCGAAGTCGGTGAGGTAACCTTTTGGAGCCAGCCGCCGAAGGTGGGACAGATGATTGGGGTGAAGTCGT

>*Priestia licheniformis* S2

TGGTTCAATCATAAAAGGTGGCTTTCAGCTACCACTTACAGATGGACCCGCGGCGCATTAGCTAGTTGGTGAGGTAACGGCTCACCAAGGCGACGATGCGTAGCCGACCTGAGAGGGTGATCGGCCACACTGGGACTGAGACACGGCCCAGACTCCTACGGGAGGCAGCAGTAGGGAATCTTCCGCAATGGACGAAAGTCTGACGGAGCAACGCCGCGTGAGTGATGAAGGTTTTCGGATCGTAAAACTCTGTTGTTAGGGAAGAACAAGTACCGTTCGAATAGGGCGGTACCTTGACGGTACCTAACCAGAAAGCCACGGCTAACTACGTGCCAGCAGCCGCGGTAATACGTAGGTGGCAAGCGTTGTCCGGAATTATTGGGCGTAAAGCGCGCGCAGGCGGTTTCTTAAGTCTGATGTGAAAGCCCCCGGCTCAACCGGGGAGGGTCATTGGAAACTGGGGAACTTGAGTGCAGAAGAGGAGAGTGGAATTCCACGTGTAGCGGTGAAATGCGTAGAGATGTGGAGGAACACCAGTGGCGAAGGCGACTCTCTGGTCTGTAACTGACGCTGAGGCGCGAAAGCGTGGGGAGCGAACAGGATTAGATACCCTGGTAGTCCACGCCGTAAACGATGAGTGCTAAGTGTTAGAGGGTTTCCGCCCTTTAGTGCTGCAGCAAACGCATTAAGCACTCCGCCTGGGGAGTACGGTCGCAAGACTGAAACTCAAAGGAATTGACGGGGGCCCGCACAAGCGGTGGAGCATGTGGTTTAATTCGAAGCAACGCGAAGAACCTTACCAGGTCTTGACATCCTCTGACAACCCTAGAGATAGGGCTTCCCCTTCGGGGGCAGAGTGACAGGTGGTGCATGGTTGTCGTCAGCTCGTGTCGTGAGATGTTGGGTTAAGTCCCGCAACGAGCGCAACCCTTGATCTTAGTTGCCAGCATTCAGTTGGGCACTCTAAGGTGACTGCCGGTGACAAACCGGAGGAAGGTGGGGATGACGTCAAATCATCATGCCCCTTATGACCTGGGCTACACACGTGCTACAATGGGCAGAACAAAGGGCAGCGAAGCCGCGAGGCTAAGCCAATCCCACAAATCTGTTCTCAGTTCGGATCGCAGTCTGCAACTCGACTGCGTGAAGCTGGAATCGCTAGTAATCGCGGATCAGCATGCCGCGGTGAATACGTTCCCGGGCCTTGTACACACCGCCCGTCACACCACGAGAGTTTGTAACACCCGAAGTCGGTGAGGTAACCTTTTGGAGCCAGCCGCCGAAGGTGGGACAGATGATTGGGGTGAAGTCGT

>*Streptomyces griseorubens* S7

GGCTCAGGACGAACGCTGGCGGCGTGCTTAACACATGCAAGTCGAACGATGAACCACTTCGGTGGGGATTAGTGGCGAACGGGTGAGTAACACGTGGGCAATCTGCCCTGCACTCTGGGACAAGCCCTGGAAACGGGGTCTAATACCGGATACTGACCCGCTTGGGCATCCAAGCGGTTCGAAAGCTCCGGCGGTGCAGGATGAGCCCGCGGCCTATCAGCTTGTTGGTGAGGTAATGGCTCACCAAGGCGACGACGGGTAGCCGGCCTGAGAGGGCGACCGGCCACACTGGGACTGAGACACGGCCCAGACTCCTACGGGAGGCAGCAGTGGGGAATATTGCACAATGGGCGAAAGCCTGATGCAGCGACGCCGCGTGAGGGATGACGGCCTTCGGGTTGTAAACCTCTTTCAGCAGGGAAGAAGCGAAAGTGACGGTACCTGCAGAAGAAGCGCCGGCTAACTACGTGCCAGCAGCCGCGGTAATACGTAGGGCGCGAGCGTTGTCCGGAATTATTGGGCGTAAAGAGCTCGTAGGCGGCTTGTCACGTCGGTTGTGAAAGCCCGGGGCTTAACCCCGGGTCTGCAGTCGATACGGGCAGGCTAGAGTTCGGTAGGGGAGATCGGAATTCCTGGTGTAGCGGTGAAATGCGCAGATATCAGGAGGAACACCGGTGGCGAAGGCGGATCTCTGGGCCGATACTGACGCTGAGGAGCGAAAGCGTGGGGAGCGAACAGGATTAGATACCCTGGTAGTCCACGCCGTAAACGGTGGGCACTAGGTGTGGGCGACATTCCACGTCGTCCGTGCCGCAGCTAACGCATTAAGTGCCCCGCCTGGGGAGTACGGCCGCAAGGCTAAAACTCAAAGGAATTGACGGGGGCCCGCACAAGCGGCGGAGCATGTGGCTTAATTCGACGCAACGCGAAGAACCTTACCAAGGCTTGACATACACCGGAAAGCATCAGAGATGGTGCCCCCCTTGTGGTCGGTGTACAGGTGGTGCATGGCTGTCGTCAGCTCGTGTCGTGAGATGTTGGGTTAAGTCCCGCAACGAGCGCAACCCTTGTCCCGTGTTGCCAGCAGGCCCTTGTGGTGCTGGGGACTCACGGGAGACCGCCGGGGTCAACTCGGAGGAAGGTGGGGACGACGTCAAGTCATCATGCCCCTTATGTCTTGGGCTGCACACGTGCTACAATGGCCGGTACAATGAGCTGCGATACCGCGAGGTGGAGCGAATCTCAAAAAGCCGGTCTCAGTTCGGATTGGGGTCTGCAACTCGACCCCATGAAGTCGGAGTCGCTAGTAATCGCAGATCAGCATTGCTGCGGTGAATACGTTCCCGGGCCTTGTACACACCGCCCGTCACGTCACGAAAGTCGGTAACACCCGAAGCCGGTGGCCCAACCCCTTGTGGGAGGGAGCTGTCGAAGGTGGGACTGGCGATTGGGACGAAGTCGT

>*Priestia endophyticus* Y5

GGCTCAGGACGAACGCTGGCGGCGTGCCTAATACATGCAAGTCGAGCGGAGTTTTGAAAAGCTTGCTTTTCAAAACTTAGCGGCGGACGGGTGAGTAACACGTGGGCAACCTGCCCTTGAGACGGGGATAACTCCGGGAAACCGGAGCTAATACCGGATAACACATATCTTCGCATGAGGATATGTTAGAAGGTGGCTTTTAGCTACCACTCAAGGATGGGCCCGCGGCGCATTAGCTAGTTGGTGAGGTAACGGCTCACCAAGGCGACGATGCGTAGCCGACCTGAGAGGGTGATCGGCCACACTGGGACTGAGACACGGCCCAGACTCCTACGGGAGGCAGCAGTAGGGAATCTTCCGCAATGGACGAAAGTCTGACGGAGCAACGCCGCGTGAGTGATGAAGGTTTTCGGATCGTAAAGCTCTGTTGTTAGGGAAGAACAAGTACCTGTTAAATAAGCAGGTACCTTGACGGTACCTAACCAGAAAGCCACGGCTAACTACGTGCCAGCAGCCGCGGTAATACGTAGGTGGCAAGCGTTGTCCGGAATTATTGGGCGTAAAGCGCGCGCAGGCGGTTCCTTAAGTCTGATGTGAAAGCCCACGGCTCAACCGTGGAGGGTCATTGGAAACTGGGGAACTTGAGTGCAGAAGAGGAGAGCGGAATTCCACGTGTAGCGGTGAAATGCGTAGAGATGTGGAGGAACACCAGTGGCGAAGGCGGCTCTCTGGTCTGTAACTGACGCTGAGGCGCGAAAGCGTGGGGAGCGAACAGGATTAGATACCCTGGTAGTCCACGCCGTAAACGATGAGTGCTAAGTGTTAGAGGGTTTCCGCCCTTTAGTGCTGCAGCAAACGCATTAAGCACTCCGCCTGGGGAGTACGGTCGCAAGACTGAAACTCAAAGGAATTGACGGGGACCCGCACAAGCGGTGGAGCATGTGGTTTAATTCGAAGCAACGCGAAGAACCTTACCAGGTCTTGACATCCTCTGCTACTTCTAGAGATAGAAGGTTCCCTTCGGGGACAGAGTGACAGGTGGTGCATGGTTGTCGTCAGCTCGTGTCGTGAGATGTTGGGTTAAGTCCCGCAACGAGCGCAACCCTTGATCTTAGTTGCCAGCATTTAGTTGGGCACTCTAAGGTGACTGCCGGTGACAAACCGGAGGAAGGTGGGGATGACGTCAAATCATCATGCCCCTTATGACCTGGGCTACACACGTGCTACAATGGATGATACAAAGGGCTGCAAAACCGCGAGGTTAAGCCAATCCCATAAAACCATTCTCAGTTCGGATTGTAGGCTGCAACTCGCCTACATGAAGCCGGAATCGCTAGTAATCGCGGATCAGCATGCCGCGGTGAATACGTTCCCGGGTCTTGTACACACCGCCCGTCACACCACGAGAGTTTGTAACACCCGAAGTCGGTGAGGTAACCTTTTGGAGCCAGCCGCCGAAGGTGGGACAGATGATTGGGGTGAAGTCGT

>*Nocardiopsis aegyptia* Y6

GGCTCAGGACGAACGCTGGCGGCGTGCTTAACACATGCAAGTCGAGCGGTAAGGCCCTTCGGGGTACACGAGCGGCGAACGGGTGAGTAACACGTGAGCAACCTGCCCCCGACTCCGGGATAAGCGGTGGAAACGCCGTCTAATACCGGATACGACCGGCCACCTCATGGTGGCTGGTGGAAAGTTTTCTCGGTTGGGGATGGGCTCGCGGCCTATCAGCTTGTTGGTGGGGTAAAGGCCTACCAAGGCGATTACGGGTAGCCGGCCTGAGAGGGCGACCGGCCACACTGGGACTGAGACACGGCCCAGACTCCTGCGGGAGGCAGCAGTGGGGAATATTGCGCAATGGGCGAAAGCCTGACGCAGCGACGCCGCGTGGGGGATGACGGCCTTCGGGTTGTAAACCTCTTTTACCACCAACGCAGGCTCCCAGTTCTCTGGGGGTTGACGGTAGGTGGGGAATAAGGACCGGCTAACTACGTGCCAGCAGCCGCGGTAATACGTAGGGTCCGAGCGTTGTCCGGAATTATTGGGCGTAAAGAGCTCGTAGGCGGCGTGTCGCGTCTGCTGTGAAAGACCGGGGCTTAACTCCGGTTCTGCAGTGGATACGGGCATGCTAGAGGTAGGTAGGGGAGACTGGAATTCCTGGTGTAGCGGTGAAATGCGCAGATATCAGGAGGAACACCGGTGGCGAAGGCGGGTCTCTGGGCCTTACCTGACGCTGAGGAGCGAAAGCATGGGGAGCGAACAGGATTAGATACCCTGGTAGTCCATGCCGTAAACGTTGGGCGCTAGGTGTGGGGACTTTCCACGGTTTCCGCGCCGTAGCTAACGCATTAAGCGCCCCGCCTGGGGAGTACGGCCGCAAGGCTAAAACTCAAAGGAATTGACGGGGGCCCGCACAAGCGGCGGAGCATGTTGCTTAATTCGACGCAACGCGAAGAACCTTACCAAGGTTTGACATCACCCGTGGACCTGCAGAGATGTGGGGTCATTTAGTTGGTGGGTGACAGGTGGTGCATGGCTGTCGTCAGCTCGTGTCGTGAGATGTTGGGTTAAGTCCCGCAACGAGCGCAACCCTTATTCCATGTTGCCAGCACGTAGTGGTGGGGACTCATGGGAGACTGCCGGGGTCAACTCGGAGGAAGGTGGGGACGACGTCAAGTCATCATGCCCCTTATGTCTTGGGCTGCAAACATGCTACAATGGCCGGTACAATGGGCGTGCGATACCGTAAGGTGGAGCGAATCCCTAAAAGCCGGTCTCAGTTCGGATTGGGGTCTGCAACTCGACCCCATGAAGGTGGAGTCGCTAGTAATCGCGGATCAGCAACGCCGCGGTGAATACGTTCCCGGGCCTTGTACACACCGCCCGTCACGTCATGAAAGTCGGCAACACCCGAAACTTGTGGCCTAACCCTTCGGGGAGGGAATGAGTGAAGGTGGGGGCTGGCGATTGGGACGAAGTCGT

# Supplementary Figures and Tables

## Supplementary Figures


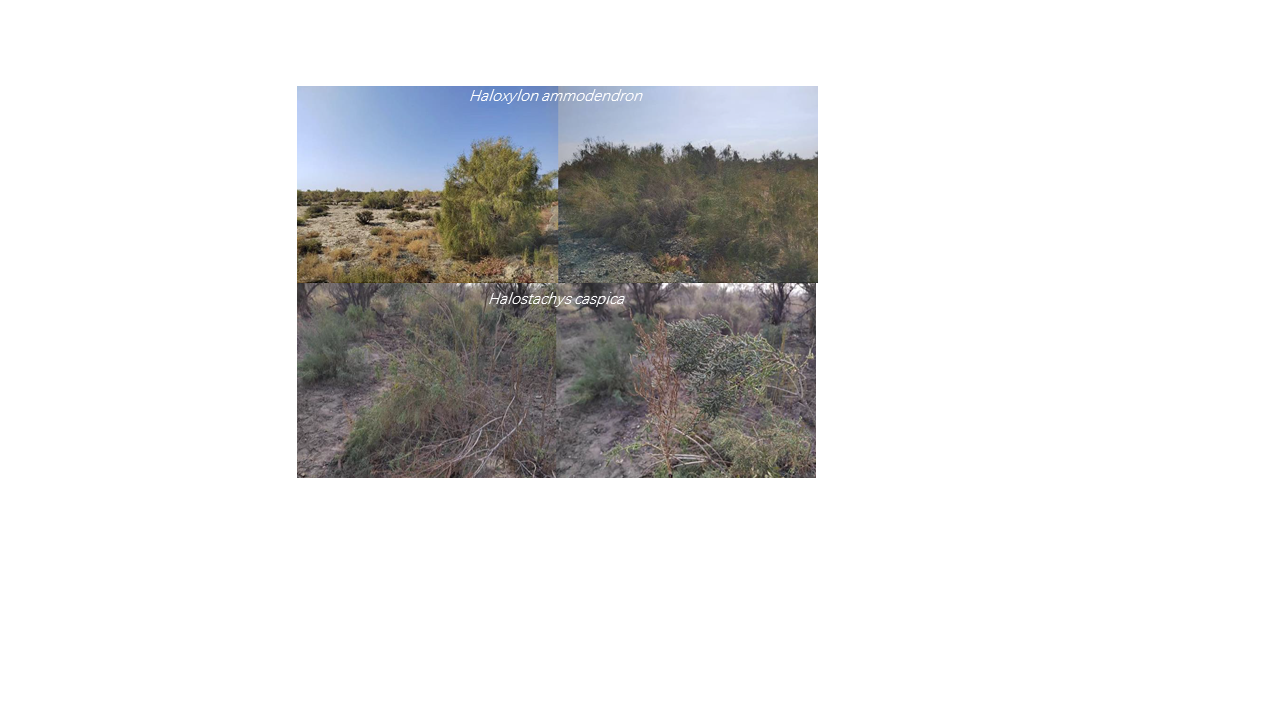


**Supplementary Figure 1**. The pictures of *Haloxylon ammodendron* and *Halostachys caspica*.


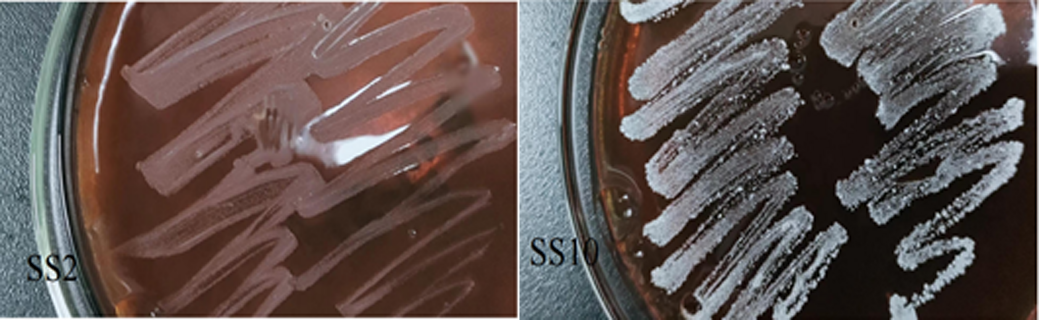


**Supplementary Figure 2**. Representive photographs of bacteria strain’s salt-alkali tolerance test (pH 12, with 2% NaCl concentration). The presence of colonies indicate bacteria can tolerate a given pH or salinity condition.


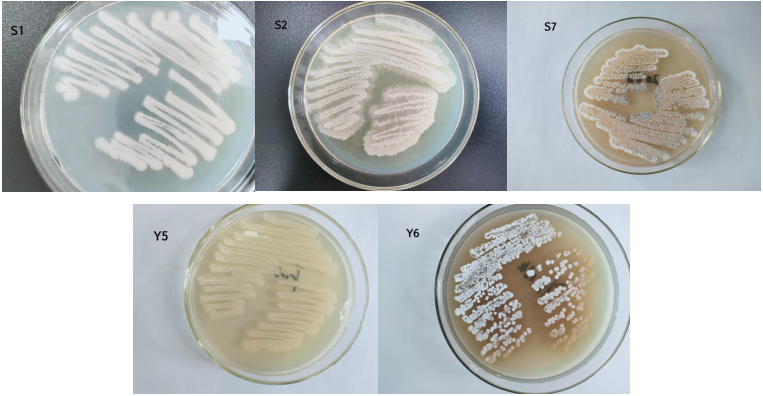


**Supplementary Figure 3.** The illustration of the plate culture of the five endophytic bacteria.


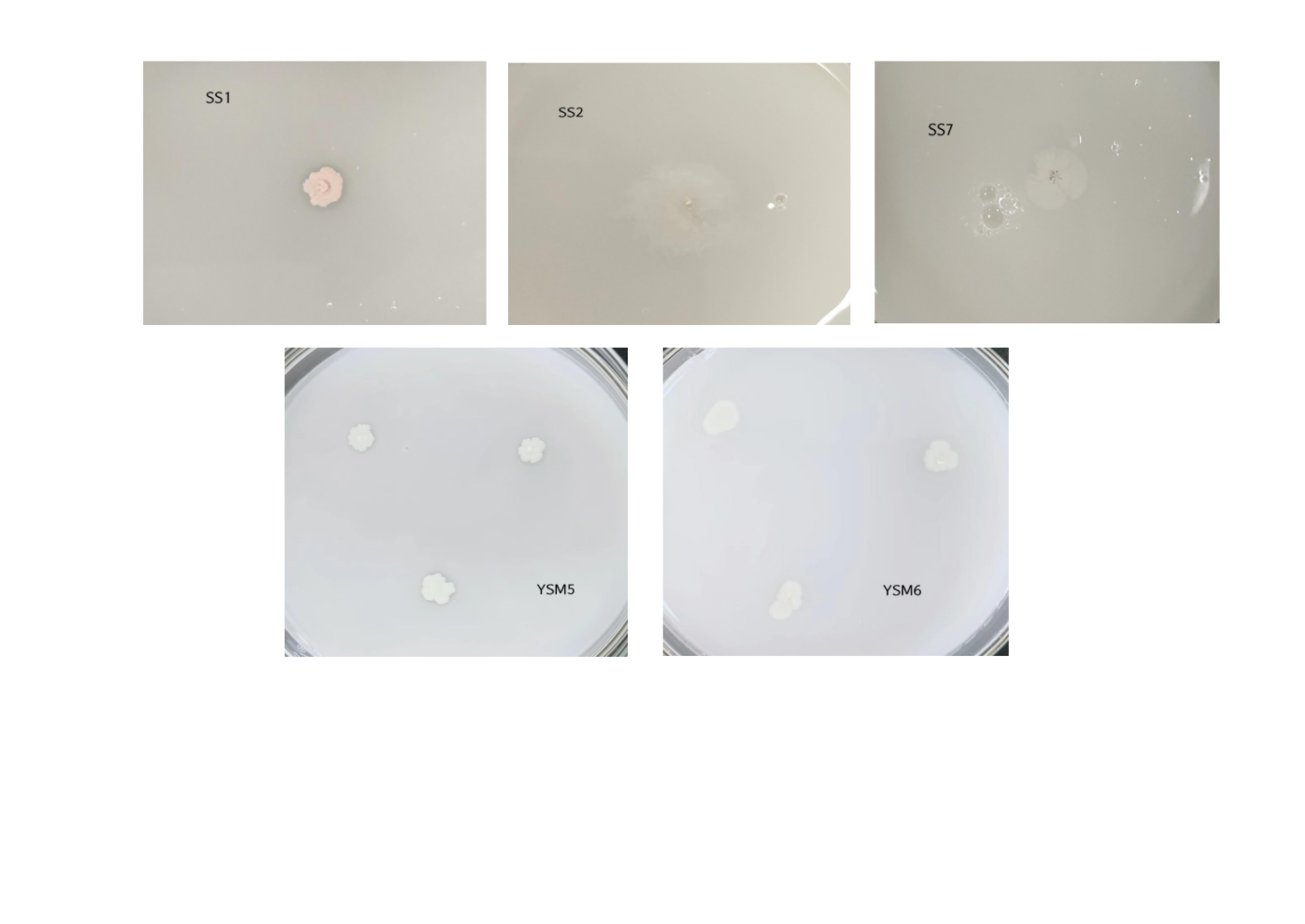


**Supplementary Figure 4.** The Nitrogenase production examination of the five endophytic bacteria. Presence of colony demonstrating nitrogen fixation ability.


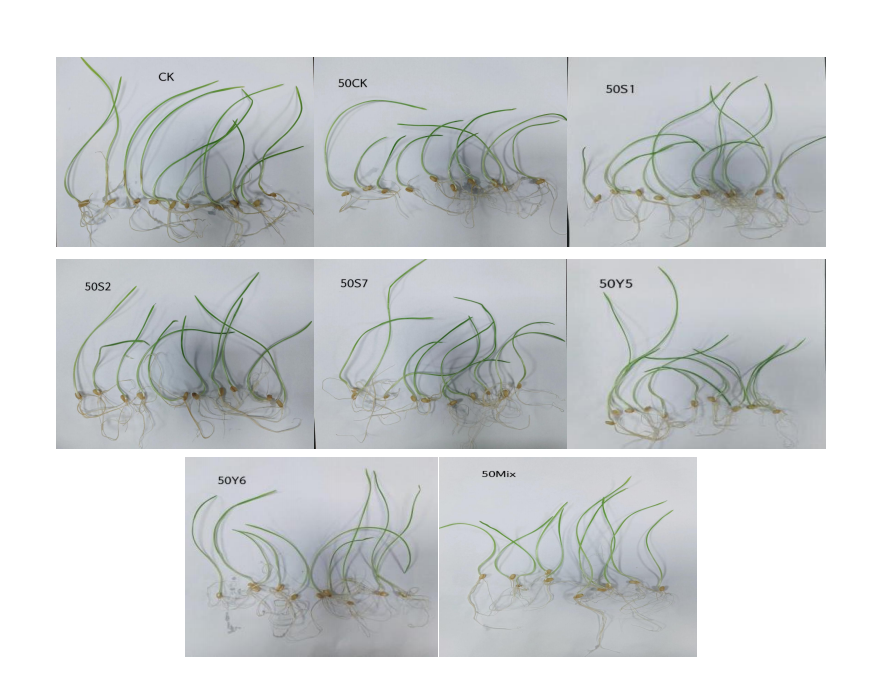


**Supplementary Figure 5**. The effects of endophytic bacteria strains on wheat seed germination under 50 mmol/L NaCl. Abbreviations: CK, untreated seeds irrigated with sterile water; 50CK, seeds treated with 50 mmol/L NaCl; 50S1, seeds inoculated with strain S1 and 50 mmol/L NaCl; 50S2, seeds inoculated with strain S2 and 50 mmol/L NaCl; 50S7, seeds inoculated with strain S7 and 50 mmol/L NaCl; 50Y5, seeds inoculated with strain Y5 and 50 mmol/L NaCl; 50Y6, seeds inoculated with strain Y6 and 50 mmol/L NaCl; 50Mix, seeds inoculated with mixed bacterial suspension and 50 mmol/L NaCl.


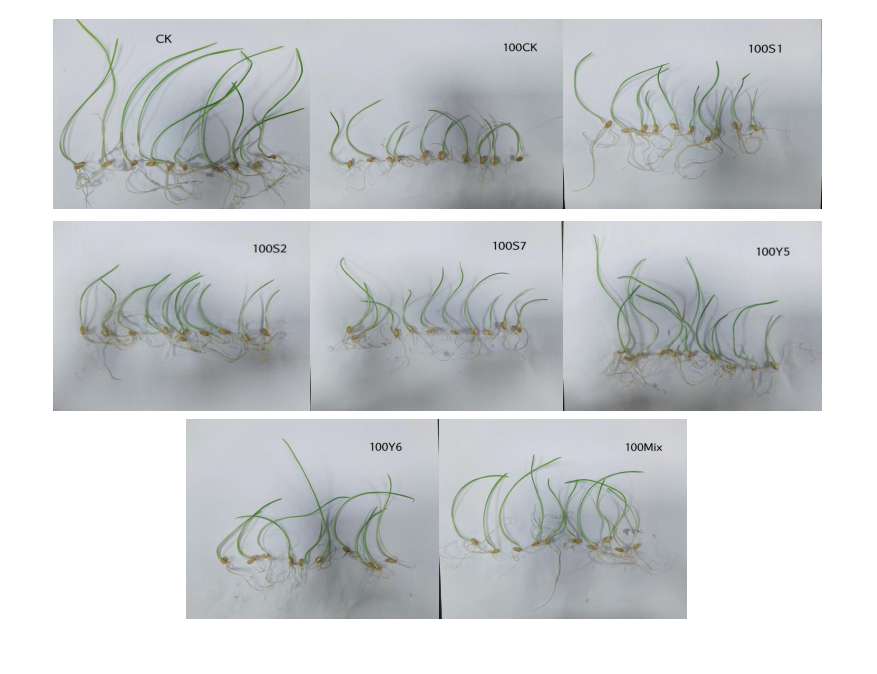


**Supplementary Figure 6**. The effects of endophytic bacteria strains on wheat seed germination under 100 mmol/L NaCl. Abbreviations: CK, untreated seeds irrigated with sterile water; 100CK, seeds treated with 100 mmol/L NaCl; 100S1, seeds inoculated with strain S1 and 100 mmol/L NaCl; 100S2, seeds inoculated with strain S2 and 100 mmol/L NaCl; 100S7, seeds inoculated with strain S7 and 100 mmol/L NaCl; 100Y5, seeds inoculated with strain Y5 and 100 mmol/L NaCl; 100Y6, seeds inoculated with strain Y6 and 100 mmol/L NaCl; 100Mix, seeds inoculated with mixed bacterial suspension and 100 mmol/L NaCl.


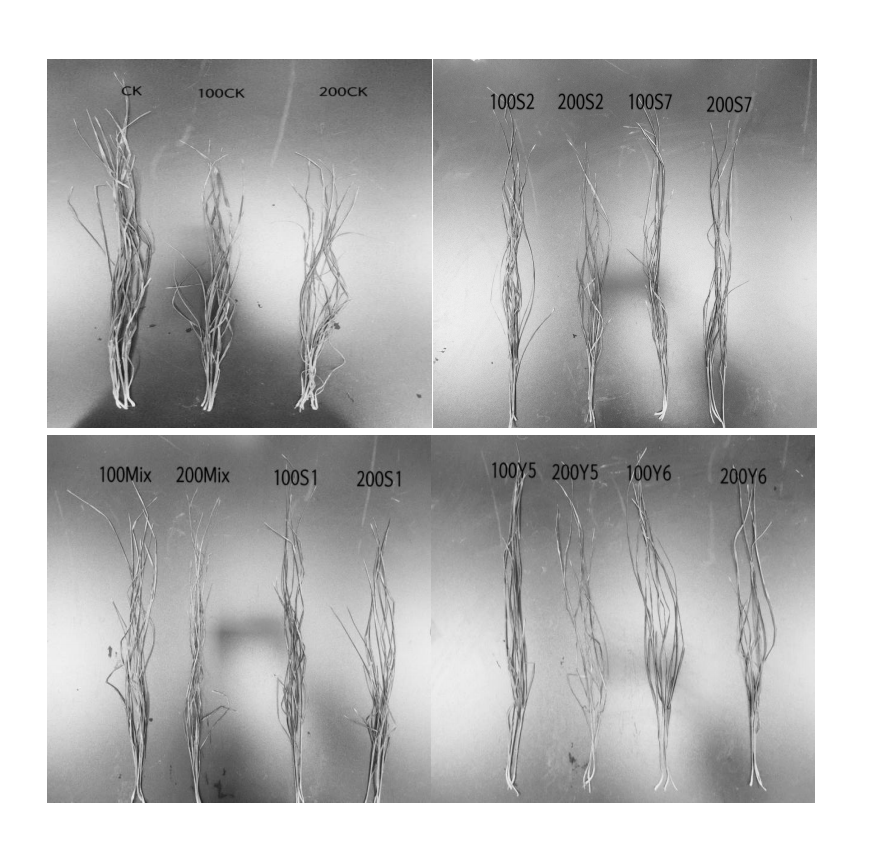


**Supplementary Figure 7**. The effects of endophytic bacteria inoculation on wheat seedlings growth under salt stress. The 100 mmol/L and 200 mmol/L NaCl treatment (100CK and 200CK) decreased wheat seedlings height compared to CK. The endophytic bacteria inoculation (single and mix) improved seedlings growth. Abbreviations: CK, untreated seedlings irrigated with sterile water; 100CK, seedlings treated with 100 mmol/L NaCl; 200CK, seedlings treated with 200 mmol/L NaCl; 100S1, seedlings inoculated with strain S1 under 100 mmol/L NaCl; 100S2, seedlings inoculated with strain S2 under 100 mmol/L NaCl; 100S7, seedlings inoculated with strain S7 under 100 mmol/L NaCl; 100Y5, seedlings inoculated with strain Y5 under 100 mmol/L NaCl; 100Y6, seedlings inoculated with strain Y6 under 100 mmol/L NaCl; 100Mix, seedlings inoculated with mixed strains under 100 mmol/L NaCl; 200S1, seedlings inoculated with strain S1 under 200 mmol/L NaCl; 200S2, seedlings inoculated with strain S2 under 200 mmol/L NaCl; 200S7, seedlings inoculated with strain S7 under 200 mmol/L NaCl; 200Y5, seedlings inoculated with strain Y5 under 200 mmol/L NaCl; 200Y6, seedlings inoculated with strain Y6 under 200 mmol/L NaCl; 200Mix, seedlings inoculated with mixed strains under 200 mmol/L NaCl.
